# Cervical cancer screening utilization and its predictors among women in bench Sheko Zone, Southwest Ethiopia: using health belief model

**DOI:** 10.1186/s12885-023-10927-x

**Published:** 2023-05-23

**Authors:** Alemayehu Sayih Belay, Wondwossen Niguse Asmare, Aychew Kassie

**Affiliations:** College of Medicine and Health Sciences, Department of Nursing, Mizan Tepi University, P.O. Box: 260, Mizan Aman, Ethiopia

**Keywords:** Cervical Cancer, Screening Utilization, Predictors, Women, Ethiopia

## Abstract

**Background:**

Cervical cancer is the fourth most frequently diagnosed and found to be the leading cause of cancer death in women, especially in low and middle-income countries. Even though cervical cancer is a preventable disease, its preventive measures have not been equitably implemented across and within different countries; especially in low and middle-income countries, due to different contributing factors.

**Objective:**

This study aimed to assess cervical cancer screening utilization and its’ predictors among Women in Bench Sheko Zone, Southwest Ethiopia.

**Method:**

A community-based cross-sectional study design was employed in Bench Sheko Zone from February 2021 to April 2021. Using a multi-stage stratified sampling method, a total of 690 women in the age range of 30–49 years were included in the study. The logistic regression analysis was used considering a 95% confidence interval and a *P*-value of < 0.05.

**Results:**

Ninety-six (14.2%) of the participants have utilized cervical cancer screening. Predictors like; age between 40–49 years (AOR = 5.35, 95% CI = [2.89, 9.90]), partner educational status of certificate and above (AOR = 4.36, 95% CI = [1.65, 11.51]), first sexual intercourse before eighteen years (AOR = 4.85, 95% CI = [2.29, 10.26]), ever used of alcohol (AOR = 3.99, 95% CI = [1.23, 12.89]), good knowledge (AOR = 8.98, 95% CI = [4.06, 19.89]), favorable attitude (AOR = 3.56, 95% CI = [1.78, 7.09]), and high perceived benefit (AOR = 2.94, 95% CI = [1.48, 5.84]) were strongly associated with cervical cancer screening utilization.

**Conclusion:**

In this study, cervical cancer screening utilization was relatively low. Therefore, promotion of the perception of women towards cervical cancer screening, and provision of health-related information towards different behavioral-related factors should have to be addressed at each level of health care.

## Background

Cervical cancer is the fourth most frequently diagnosed cancer and leading cause of cancer death in women, with an estimation of 604,000 new cases and 342,000 deaths worldwide in 2020 [1]. Of estimated new cases and deaths, 117,316 (10.6%) new cases and 76,745(10.8%) death of cervical cancer occur in Africa every year. Yet, because of poor access to screening and treatment services, the vast majority of deaths occur in women living in low- and middle-income countries (LMICs) [2]. In Ethiopia, 7,445 (9.6%) new cases and 5,338 (10.3%) death of cervical cancer occur every year [3].

Cervical cancer is contributed by different factors including; human papillomavirus (HPV) [4], some sexually transmittable infections (human immunodeficiency virus (HIV) [5], herpes simplex virus type-2 [6], chlamydia [7], and gonorrhea [8], smoking [9], a higher number of childbirths [10], multiple sexual partners [11], poor socio-economical status [12], and long-term use of oral contraceptives [13], and corticosteroid drugs [14].

Cervical cancer is a preventable disease. As a result, it can be prevented using comprehensive cervical cancer control methods which include primary prevention (vaccination against HPV), secondary prevention (screening and treatment of pre-cancerous lesions), tertiary prevention (diagnosis and treatment of invasive cervical cancer), and palliative care [4, 15]. However, these preventive measures have not equally implemented across and within countries. In low and middle-income countries (LMICs), only 44% of women have screened for cervical cancer, with the lowest prevalence among women in Sub-Saharan Africa, compared with > 60% of women who have screened in high-income countries [15]. In 2015, the cervical cancer screening rate in Ethiopia is extremely low 2.9% [16].

High-quality screening programs are also important to prevent cervical cancer among unvaccinated women. The world health organization (WHO) recommends the screening of women aged 30 to 49 years either through visual inspection with acetic acid (VIA) in low-resource settings or a papanicolaou test (cervical cytology) every 3 to 5 years or HPV testing every 5 years, coupled with timely and efficacious treatment of precancerous lesions [17, 18].

In Ethiopia, the first cancer prevention and control plan was published in 2015 [19], and a “screen-and-treat” approach has been implemented using visual inspection with acetic acid (VIA) and cryotherapy which were found to be feasible and appropriate screening and treating methods [20]. Despite the implementation of this approach, the cervical cancer screening utilization remains low [21–25] due to different factors like; low knowledge [26], poor attitude [27], inadequate diagnostic facilities [28], poorly structured referral system [29], financial constraints [30], poor infrastructure [31], young women [32], and low parity [32]. Due to these reasons, the incidence and mortality of women associated with cervical cancer remain increasing [3]. However, the incidence and mortality related to cervical cancer have declined markedly in developed countries like; United States and England due to a widespread screening practice [33, 34].

Thus, to increase the cervical cancer screening utilization among eligible women, it is necessary to identify factors using a health belief model (HBM) which helps to predict the health behaviors of women [35, 36]. However, there is paucity of studies related to the perception of women towards cervical cancer and its screening in Ethiopia. Therefore, this study aimed to assess cervical cancer screening utilization and associated factors among women in Bench Sheko Zone, Ethiopia.

## Method and materials

### Study design and period

A community based cross-sectional study design was employed from February 2021 to April 2021.

### Study setting

The study was conducted in Bench Sheko Zone, which is found in the Southern Nation Nationality and Peoples’ Region (SNNPR) State, Ethiopia. Bench Sheko is bordered on the west by South Sudan, on the north by Sheka Zone, on the northwest by Keffa Zone, and on the east by west Omo Zone. The administrative center of Bench Sheko Zone is Mizan Teferi, which is located 561 km far from Addis Ababa. The zone has a total of two administrative towns and six districts namely; Debub Bench, Guraferda, Semen Bench, Shey Bench, Gide Bench, Sheko, Cize town administration, and Mizan Aman town administration with a total population of 609,588 (2011 Zonal Report). In Bench Sheko Zone, there are 1 teaching hospital, 1 primary hospital, 25 health centers, and 130 health posts with different health services. Currently, only the Mizan Tepi University Teaching hospital and Mizan health center give cervical cancer screening service.

### Study participants

According to Ethiopian cervical cancer prevention and control guideline [37], all women aged between 30–49 years in Bench Sheko Zone were source population. Hence, women aged between 30–49 years and who lived in selected kebeles during the study period were the study population. Furthermore, women in the age range of 30–49 years and who lived for at least 6 months in the study area were included in the study, whereas, those who were critically ill during the data collection period were excluded.

### Sample size determination

The sample size was calculated using a single population proportion formula of $n=({z\frac{a}{2})}^{2}\frac{p(1-p)}{{d}^{2}}$, where n = the sample size, d = degree of precision = 0.04, Z α/2 = 1.96 by assuming 95% confidence interval and the population proportion of 0.155 (15.5%), as it was used for the study conducted in Jimma town, Southwest Ethiopia [22]. Then, by adding a 10% non-response rate and design effect of 2, the final sample size was 690.

### Sampling procedure

For this particular study, a multi-stage stratified sampling technique was used to select the study participants. The primary sampling units, the 3 districts; Shey Bench, Gidi Bench, and Debub Bench, and 1 administrative town, Mizan Aman were selected by simple random sampling from the total of 6 districts and two administrative towns in Bench Sheko Zone, respectively.

**Fig. 1:**
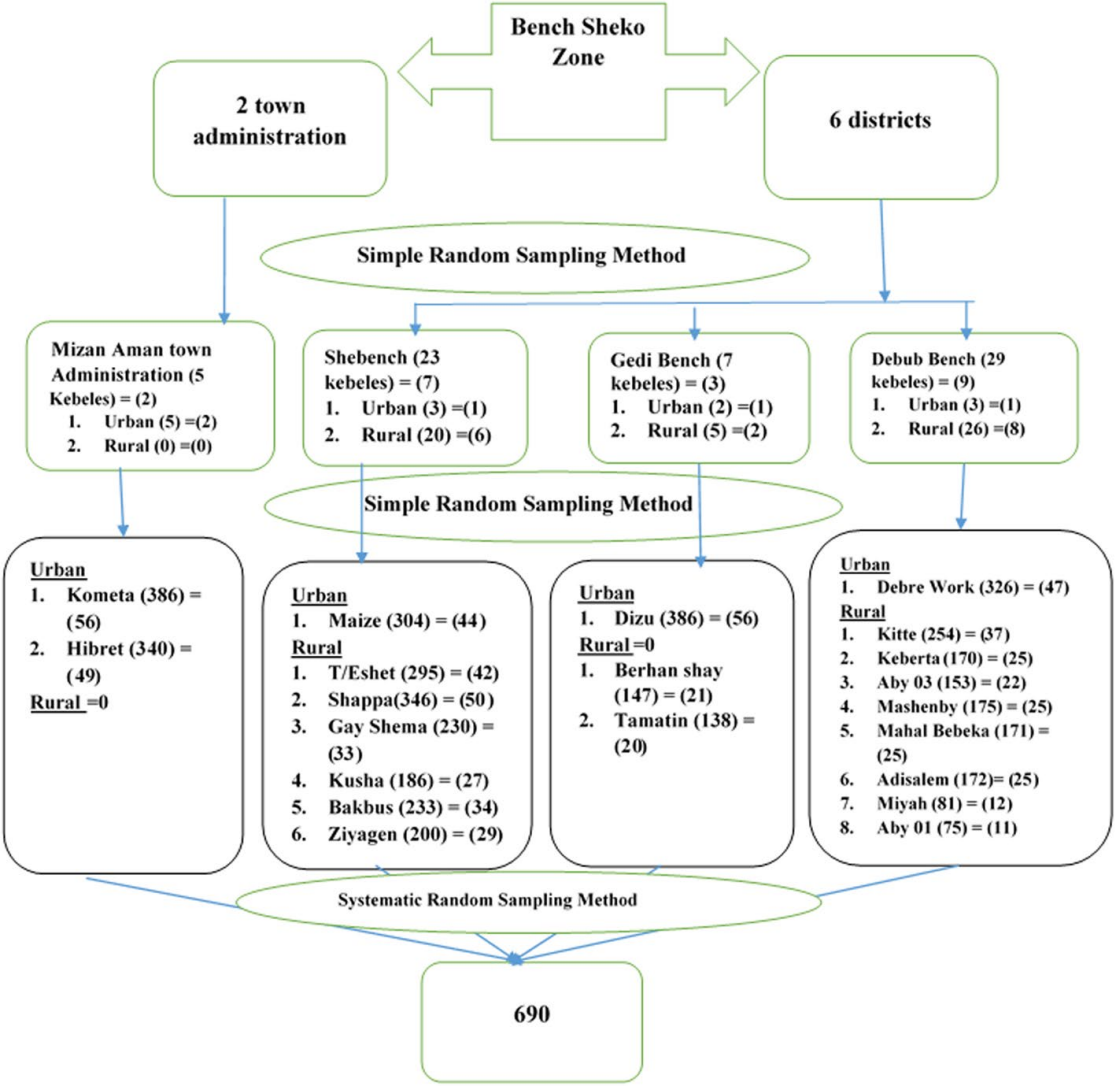
Schematic presentation of sampling procedure for study on cervical cancer screening utilization among women in Bench Sheko Zone, 2021

The secondary sampling units, the 21 kebeles (the smallest administrative unit within the district) were randomly selected from each stratum (urban and rural). Then, the sample size was distributed to each selected kebeles by population proportion to size (PPS) formula. Then, the tertiary sampling units, the households were selected by using systematic random sampling. The sampling interval (k^th^ interval) of six was used to select the eligible participants using the household lists which were determined after the survey. Then, the next household was selected systematically with a random starting point which was selected from the first 6 households, and the next eligible woman in the household was interviewed every 6^th^ household. For households with more than one eligible woman, only one study participant was selected using the lottery method. Revisit of three times was made when the eligible respondent was not available at the time of the data collection [Fig. 1].

### Operational definitions

#### Cervical cancer screening utilization

Was defined as a woman who ever utilize cervical cancer screening within the past 5 years [25, 38].

#### Multiple sexual partners

Those women who have ever had more than one sexual partner in their life serially or at the same time [39].

#### Knowledgeable

Women who were responded to the knowledge-related questions and a score of the mean value or above were considered as knowledgeable [25, 40].

#### Favorable attitude

Women who were responded to the attitude-related questions and a score of the mean value or above were considered to have a favorable attitude [25, 40].

#### Perceived susceptibility for cervical cancer

Perception of women about the chances of experiencing a risk or acquiring cervical cancer [22, 41, 42].

#### Perceived severity of cervical cancer

Perceptions of women on the seriousness of contracting cervical cancer [22, 42, 43].

#### The perceived benefit of undergoing cervical cancer screening

Refers to the perception of the positive consequences that are caused by the utilization of cervical cancer screening [22, 42, 44].

#### Perceived barriers for undergoing cervical cancer screening

Refers to the perceptions of women on the obstacles or factors to undergoing cervical cancer screening [22, 42, 45].

Perceived susceptibility, severity, benefit, and barriers were assessed using the 5 points Likert Scale (1: strongly disagree, 2: Disagree, 3: Neutral, 4: Agree, 5: strongly agree). The total scores were computed and dichotomized into high/positive and low/negative using the mean score [22].

### Data collection tools and procedure

A structured interviewer administered questionnaire was used to collect the data which was adapted from different reviewed literature [22, 24, 25, 38, 46–48]. The questionnaire was translated from English to the national language Amharic and to assure its consistency, it was translated back to English by another linguistic professional. The data were collected by eight trained BSc nurses and supervised by four public health professionals. Before doing the actual data collection, three days of training was given regarding the objective of the study on the data collection tool, interviewing approach, and privacy and confidentiality. The questionnaire was also pretested among 5% of the total study participants in Sheka Zone.

### Data quality assurance

The quality of the data was assured by providing three days training for data collectors and using of pretested questionnaire. Moreover, close supervision of the data collection process was done and the questionnaire was checked for consistency, clarity, and completeness.

### Data processing and analysis

After data collection was completed, the data was entered into Epi-data manager version 4.0.2.101 and exported to Statistical Package for the Social Sciences (SPSS) version 22.0 for analysis. Descriptive statistics like frequency, percentage, and mean with standard deviation were used. Bivariate logistic regression analysis was performed to select variables for multivariate analysis with a 95% confidence interval and a p-value less than 0.05. Finally, multivariate logistic regression analysis was performed and a p-value of less than 0.05 was considered as strong predictor variables for cervical cancer screening utilization.

## Results

### Socio-demographic characteristics of the respondents

From the total of 690 eligible women, 678 women have participated in the study yielding a response rate of 98.2%. The mean ± SD age of the participants was 36.01 (SD ± 5.11) years. More than half, 415 (61.2%) of the participants were urban residents, and 297 (43.8%) were protestant religion followers. From the total participants, more than three-fourth, 527(77.7%) of them were currently married. Concerning the respondents’ educational status, 188 (27.7%) of them were unable to read and write. Of all participants, 265(39.1%) were housewives, 194(28.6%) had partners who able to read and write, and 195(28.8%) had partners whose occupations were merchants [Table 1].

**Table 1:** Socio-demographic characteristics of respondents related to cervical cancer screening utilization in Bench Sheko Zone, Southwest Ethiopia, 2021

Variables	N (%)
**Place of residence**	
Urban	263(38.8)
Rural	415(61.2)
**Age of respondents**	
30–39	500(73.7)
40–49	178(26.3)
Mean ± SD	36.01 (SD ± 5.11)
**Marital status**	
Married	527(77.7)
Single	63(9.3)
Divorced	54(8.0)
Widowed	34(5.0)
**Religion**	
Protestant	297(43.8)
Orthodox	260(38.3)
Muslim	99(14.6)
Other *	22(3.2)
**Ethnicity**	
Bench	221(32.6)
Kefa	199(29.4)
Amhara	142(20.9)
Sheka	35(5.2)
Tigre	45(6.6)
Oromo	36(5.3)
**Respondent ‘s educational status**	
Unable to write and read	188(27.7)
Able to read and write	148(21.8)
Primary (1–8) grade	122(18.0)
Secondary (9–12) grade	135(19.9)
Certificate and above	85(12.5)
**Respondent ‘s occupational status**	
Housewife	265(39.1)
Merchant	102(15.0)
Student	123(18.1)
Farmer	70(10.3)
Government employee	71(10.5)
Daily worker	47(6.9)
**Partner occupational status**	
Unable to write and read	169(24.9)
Able to read and write	194(28.6)
Primary (1–8) grade	126(18.6)
Secondary (9–12) grade	101(14.9)
Certificate and above	88(13.0)
**Partner occupational status**	
Farmer	177(26.1)
Merchant	195(28.8)
Gov't Employee	109(16.1)
Student	83(12.2)
Daily worker	75(11.1)
NGO	39(5.8)
**Monthly household income in Ethiopian Birr (ETB)**	
Median (IQR)	6000(5000–9000)
**Total**	678 (100%)

^a^Catholic, *IQR* Inter Quartile Range

### Reproductive health and behavioral related characteristics of the respondents

From the total participants, more than two-third, 470(69.3%) of them had the first sexual intercourse before the age of eighteen, whereas, majority or 635(93.7%) of them gave their first child after the age of eighteen with the mean ± SD of 21.8(SD ± 2.6) years. Of all the study participants, about 155(22.9%), 98(14.5%), and 81(11.9) had a history of HIV test, and a history of sexual transmitted diseases (STD), and smoking history, respectively. Concerning contraceptive history, about 427(63.0) of the participants were used modern contraceptives [Table 2].

**Table 2:** Reproductive health and behavioral characteristics of the respondents related to cervical cancer screening utilization in Bench Sheko Zone, Southwest Ethiopia, 2021

Variables	N (%)
**Age of first sexual intercourse**	
< 18	470(69.3)
> = 18	208(30.7)
Mean ± SD	17.6(SD ± 2.08)
**Age at first child birth**	
< 18	43(6.3)
> = 18	635(93.7)
Mean ± SD	21.8(SD ± 2.6)
**History of HIV test**	
Yes	155(22.9)
No	523(77.1)
**History of STD**	
Yes	98(14.5)
No	580(85.5)
**Family history of cervical cancer**	
Yes	25(3.7)
No	653(96.3)
**Multiple sexual partner**	
Yes	334(49.3)
No	344(50.7)
**Ever use of modern FP method**	
Yes	427(63.0)
No	251(37.0)
**History of smoking**	
Yes	81(11.9)
No	597(88.1)
**Ever use of alcohol**	
Yes	106(15.6)
No	572(84.4)
**Safe condom utilization**	
Yes	71(10.5)
No	607(89.5)

### Cervical cancer screening utilization

**Fig. 2:**
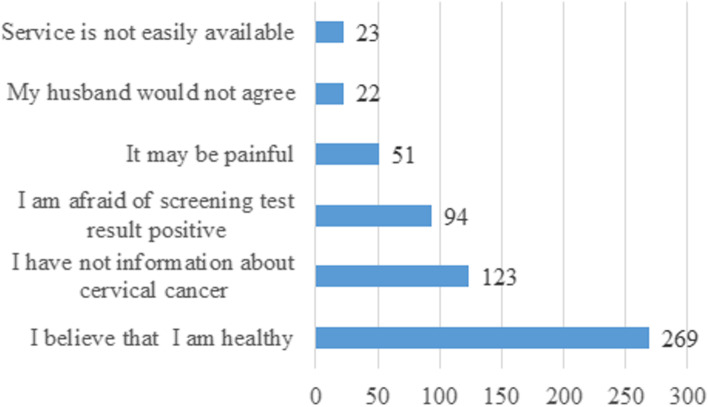
Reasons of declining for cervical cancer screening among women in Bench Sheko Zone, Southwest Ethiopia, 2021

Ninety-six (14.2%) (95% CI: 11.5, 16.8) of the participants have ever utilized cervical cancer screening at least once in the last five years. Among the total participants who screened for cervical cancer, about 56 (8.3%), 25 (3.7%), and 15(2.2%) of them were screened due to the offer by the health professionals, self-initiated, and know someone screened, respectively. The most common reason for the participants not to utilize cervical cancer screening was feeling healthy, 269 (39.7%) [Fig. 2].

### Predictors of cervical cancer screening utilization

Using bivariate logistic regression, different factors like; the age of participants, partner educational status, age at first sex, ever use of alcohol, smoking history, overall knowledge, overall attitude, perceived susceptibility, perceived severity, and perceived benefit were found to be significantly associated with cervical cancer screening utilization.

After adjusting for possible confounding factors using multivariate logistic regression model; the age of participants, partner educational status, age at first sex, ever use of alcohol, overall knowledge, overall attitude, and perceived benefit were found to be strong predictors of cervical cancer screening utilization.

Participants with the age range of 40–49 years were five times more likely to use cervical cancer screening than those whose ages were 30–39 years (AOR = 5.35, 95% CI = [2.89, 9.90]). Participants with partner educational status of the certificate and above were four times more likely to use cervical cancer screening than those with partner educational status of unable to read and write (AOR = 4.36, 95% CI = [1.65, 11.51]). Those who had first sexual intercourse before eighteen years were almost five times more likely to use cervical cancer screening than those who had first sexual intercourse after eighteen (AOR = 4.85, 95% CI = [2.29, 10.26]). Participants who had ever used alcohol were almost four times more likely to use cervical cancer screening than their counterparts (AOR = 3.99, 95% CI = [1.23, 12.89]). Knowledgeable participants were almost nine times more likely to use cervical cancer screening than their counterparts (AOR = 8.98, 95% CI = [4.06, 19.89]). Those who had a favorable attitude were almost four times more likely to use cervical cancer screening than their counterparts (AOR = 3.56, 95% CI = [1.78, 7.09]). Having perceived benefit of cervical cancer screening utilization were almost four times more likely to use cervical cancer screening than their counterparts (AOR = 2.94, 95% CI = [1.48, 5.84]) [Table 3].

**Table 3:** Predictors associated with cervical cancer screening utilization among women in Bench Sheko Zone, Southwest Ethiopia, 2021

Variables	Cervical Cancer Screening Utilization		COR (95% CI)	AOR (95% CI)
	No N (%)	Yes N (%)		
**Age of participant (in year)**				
30–39	448(89.6)	52(10.4)	1.00^a^	1.00^a^
40–49	134(75.3)	44(24.7)	2.83(1.81–4.42)	5.35(2.89–9.90)*
**Partner Educational Status**				
Unable to read and write	158(93.5)	11(6.5)	1.00^a^	1.00^a^
Able to read and write	169(87.1)	25(12.9)	2.13(1.01–4.46)	2.04(.830–5.02)
Primary (1–8)	106(84.1)	20(15.9)	2.71(1.25–5.89)	2.53(.99–6.455)
Secondary (9–12)	82(81.2)	19(18.8)	3.33(1.51–7.33)	4.06(1.47–11.17)*
Certificate and above	67(76.1)	21(23.9)	4.50(2.06–9.86)	4.36(1.65–11.51)*
**Age at First Sex**				
< 18	386(82.1)	84(17.9)	3.55(1.89–6.67)	4.85(2.29–10.26)*
> = 18	196(94.2)	12(5.8)	1.00^a^	1.00^a^
**Ever use of alcohol**				
Yes	65(61.3)	41(38.7)	5.93(3.67–9.58)	3.99(1.23–12.89)*
No	517(90.4)	55(9.6)	1.00^a^	1.00^a^
**History of smoking**				
Yes	46(56.8)	35(43.2)	6.69(4.00–11.17)	2.42(.69–8.472)
No	536(89.8)	61(10.2)	1.00^a^	1.00^a^
**Overall knowledge**				
Not knowledgeable	256(95.9)	11(4.1)	1.00^a^	1.00^a^
Knowledgeable	326(79.3)	85(20.7)	6.07(3.17–11.6)	8.98(4.06–19.89)*
**Overall attitude**				
Unfavorable attitude	232(93.2)	17(6.8)	1.00^a^	1.00^a^
Favorable attitude	350(81.6)	79(18.4)	3.08(1.78–5.34)	3.56(1.78–7.09)*
**Perceived susceptibility**				
Low	311(89.9)	35(10.1)	1.00^a^	1.00^a^
High	271(81.6)	61(18.4)	2.00(1.28–3.13)	1.51(.79–2.86)
**Perceived severity**				
Low	275(91.4)	26(8.6)	1.00^a^	1.00^a^
High	307(81.4)	70(18.6)	2.41(1.49–3.89)	1.09(.56–2.15)
**Perceived benefit**				
Low	221(92.5)	18(7.5)	1.00^a^	1.00^a^
High	361(82.2)	78(17.8)	2.65(1.55–4.55)	2.94(1.48–5.84)*

^*^Adjusted for all significant variables *p* < 0.05

^a^ = Reference Category

*CI* Confidence interval

## Discussion

Cervical cancer is a public health problem worldwide, and it is one of the leading causes of death in women [18]. The WHO and Ethiopian Federal Ministry of Health (FMOH) recommends cervical cancer screening for age-eligible women every three to five years [19, 49]. This approach enables for the decrement of the incidence and mortality of women associated with cervical cancer [33]. Therefore, this community-based study was conducted to assess the cervical screening utilization among women in Bench Sheko Zone, Ethiopia.

Hence, in this study, the cervical cancer screening utilization was 14.2%. This result was found to be low as compared with the studies conducted in Uganda, 30.3% [50], Nigeria, 20.6% [51], Tanzania, 22% [52] and in other parts of Ethiopia like; Wolatita, 22.9% [53], Addis Ababa, 25% [54], and Gondar, 23.5% [55]. The possible reasons could be due to limited access to screening services, the difference in socio-demographic characteristics and the difference in study subjects. For instance, the study subjects in the study conducted in Uganda and Gondar, Ethiopia were all HIV-infected women.

n contrast to the other studies, this study was found to be higher in the cervical cancer screening utilization compared with the studies conducted in different areas where, 0.2% in Ghana [56], 5.4% in Debre Markos, Northwest Ethiopia [25], and 5.9% in Arbaminch town, Southern Ethiopia [57]. This difference might be attributed to the difference in the study population, the difference in the study period and area, and the difference in the status of knowledge and attitude of the study population.

In this study, women with advanced age were more likely to use cervical cancer screening than young age women. This finding was supported by the other studies conducted in Malawi [58], and in other parts of Ethiopia like; Mekelle [59], Dire Dawa [26], and Arbaminch [57]. The plausible explanation for this might be due to the fact that the advanced age of women is believed to be a risk factor for cervical cancer where this might lead the woman for the repeated visit of health care services. Moreover, women with advanced age might increase the chance of accessing health-related information related to cervical cancer and its screening services which in turn leads them to utilize cervical cancer screening services. In women with advanced age, high parity is also common which is known to be a risk factor for cervical cancer [60] and this might also contribute for cervical cancer screening.

Participants with high partner educational status were more likely to use cervical cancer screening than those with partner educational status of unable to read and write. This finding was found to be consistent with the studies conducted in Ghana [61], Kenya [62] and in different parts of Ethiopia like; Jimma [22], Debre Markos [25], and Shabadino [21]. This could be due to the fact that educated women have access to different health-related information including the severity of cervical cancer and the benefit of cervical cancer screening utilization.

In this study, the first sexual intercourse before eighteen years was also found to be a strong predictor variable for cervical cancer screening utilization. This finding is consistent with the study conducted in Debre Markos [25]. The possible explanation for this might be women who started sexual intercourse at an early age, may have increased lifetime sexual partners which in turn increase the chance of being infected with sexually transmitted infection (STI) with its signs and symptoms which lead to visit health facilities.

Ever use of alcohol was found to be associated with cervical cancer screening. This finding is in line with the study conducted in USA [63]. These findings give evidence to the idea that alcoholic women are at higher risk for the progression from human papillomavirus infection to the in situ cervical cancer and invasive cervical cancer [64]. This health constraint of women will probably enable them to visit health institutions for cervical cancer screen utilization.

The good knowledge status of the participants was more likely to use cervical cancer screening than their counterparts. This result was congruent with different studies conducted in Kenya [65], Jamaica [32], Tanzania [39], Japan [66], China [67], Dire Dawa [26] and, Finote Selam, Ethiopia [68]. This may be due to the fact that women with good knowledge about cervical cancer will have a better understanding of risk factors, clinical manifestations, complications, and prevention and treatment options, for instance; cervical cancer screening procedure and cryotherapy. Therefore, knowledge on cervical cancer will contribute for the good practice of cervical cancer screening utilization.

Participants with favorable attitudes were found to be associated with cervical cancer screening utilization. Similarly, other studies conducted in Ethiopia, like in Hosanna [69], Mekelle [59], Gondar [55], and Finote Selam [68] also support our finding. This could be explained by that the women with favorable attitudes will have good perceptions (perceived benefit, perceived susceptibility, perceived severity, and perceived barrier) towards the cervical cancer prevention and control mechanisms which imposes the women to engage in cervical cancer screening utilization [70].

Furthermore, in this study, women with high perceived benefits were more likely to use the screening service. Similarly, this is supported by the study conducted in Arsi, Southeastern Ethiopia [71]. A possible explanation for this might be the perception towards the positive consequences secondary to the utilization of cervical cancer screening will help the women to be aware of the procedure properly, and it will also direct them to be engaged in cervical cancer screening utilization.

This study has its own limitations. First, since cross-sectional study design was implemented, it can’t establish a cause-effect relationship between the independent variables and dependent variables (cervical cancer screening utilization). Second, behavioral characteristics like alcohol utilization were not measured for its concentration of different types of alcohol. Despite these limitations, the result of this study may provide important insight for the policymakers and concerned bodies at different health sectors.

## Conclusion

In this study, the cervical cancer screening utilization was relatively low. Different predictors like; the age of participants, partner educational status, age at first sex, ever use of alcohol, overall knowledge, overall attitude, and perceived benefit were found to be strong predictors of cervical cancer screening utilization. Therefore, promotion of the perception of women towards cervical cancer screening, and provision of health-related information towards different behavioral-related factors should have to be addressed at each level of health care.

## Data Availability

All relevant data are included within the manuscript, but any additional data required are available from the primary author, [Alemayehu Sayih Belay], upon request. Email: Alex.sayihalem2018@gmail.com.

## Abbreviations

AOR: Adjusted Odd Ratio
CI: Confidence Interval
COR: Crude Odd Ratio
ETB: Ethiopian Birr
FMOH: Federal Ministry of Health
HIV: Human Immunodeficiency Virus
HPV: Human Papillomavirus
IQR: Inter Quartile Range
LMICs: Low- and Middle-Income Countries
PPS: Population Proportion to Size
SD: Standard Deviation
SPSS: Statistical Package for The Social Sciences
STD: Sexual Transmitted Disease
VIA: Visual Inspection with Acetic Acid
WHO: World Health Organization
